# Time-dependent deacclimation after cold acclimation in *Arabidopsis thaliana* accessions

**DOI:** 10.1038/srep12199

**Published:** 2015-07-15

**Authors:** Ellen Zuther, Ilona Juszczak, Yang Ping Lee, Margarete Baier, Dirk K. Hincha

**Affiliations:** 1 Max-Planck-Institut für Molekulare Pflanzenphysiologie, Am Mühlenberg 1, D-14476 Potsdam, Germany; 2 FU Berlin, Institute of Biology, DCPS, Plant Physiology, Königin-Luise-Straße 12-16, 14195 Berlin, Germany

## Abstract

During low temperature exposure, *Arabidopsis thaliana* and many other plants from temperate climates increase in freezing tolerance in a process termed cold acclimation. However, the correct timing and rate of deacclimation, resulting in loss of freezing tolerance and initiation of growth is equally important for plant fitness and survival. While the molecular basis of cold acclimation has been investigated in detail, much less information is available about deacclimation. We have characterized the responses of 10 natural accessions of *Arabidopsis thaliana* that vary widely in their freezing tolerance, to deacclimation conditions. Sugar, proline and transcript levels declined sharply over three days in all accessions after transfer of cold acclimated plants to ambient temperatures, while freezing tolerance only declined in tolerant accessions. Correlations between freezing tolerance and the expression levels of *COR* genes and the content of glucose, fructose and sucrose, as well as many correlations among transcript and solute levels, that were highly significant in cold acclimated plants, were lost during deacclimation. Other correlations persisted, indicating that after three days of deacclimation, plant metabolism had not completely reverted back to the non-acclimated state. These data provide the basis for further molecular and genetic studies to unravel the regulation of deacclimation.

Cold acclimation, the increase in freezing tolerance during exposure to low, but non-freezing temperatures, is a common response of plants from temperate climate zones to seasonal low temperature^1^. Cold acclimation in any given species is a genetically complex process, involving a wide range of physiological changes that are to a large extent based on equally complex changes in signal transduction and gene expression (see e.g.^2^,^3^,^4^,^5^ for reviews). Adding to this complexity, it has been estimated that almost half of all angiosperm families have members that are able to cold acclimate and that this trait has developed several times independently^1^.

However, cold acclimation is only one, albeit the most thoroughly investigated, aspect of successful winter survival. Of equal importance for plant fitness and survival is the correct timing and rate of the loss of freezing tolerance (deacclimation) in spring when plants undergo the transition to growth and development (see^6^ for a recent review). If this transition is made too late, the plants lose valuable time during the growth season, while a premature transition involves the danger of freezing damage during a late-season cold spell. This situation is strongly influenced by the effects of global climate change. Due to global warming, winters in the cold regions of the Earth are getting milder and therefore mid-winter maximum freezing tolerance may become less important in the future for plant survival and geographical distribution, and also for crop yields. However, higher spring temperatures can result in early commencement of plant development^6^ that leads to premature deacclimation. This may result in catastrophic freezing damage to the vegetation of large geographical areas^7^,^8^ with negative impacts on global ecosystem^9^ and agricultural productivity^10^. The frequency of such climatic constellations is predicted to increase with further climate change^11^.

In spite of the obvious ecological and agronomic importance of the deacclimation process, research in this area is lagging far behind the level of understanding that has been achieved for cold acclimation. While there is a considerable number of ecological studies on the phenology and geographical distribution of trees in relation to deacclimation (reviewed in^6^), only a limited number of physiological and gene expression studies has been reported and a molecular understanding of the process of deacclimation and its regulation is completely lacking (see^12^,^13^ for reviews).

Since the majority of molecular studies on cold acclimation have been performed with *Arabidopsis thaliana* (see^14^,^15^,^16^ for reviews), this species is an obvious choice to also investigate molecular aspects of deacclimation, but to date only one such study has been published, which is limited to transcript data in the accession Col-0^17^. Natural variation in freezing tolerance and cold acclimation has been characterized extensively in different accessions of Arabidopsis^18^,^19^,^20^,^21^,^22^,^23^, allowing direct extension of this approach to the investigation of deacclimation mechanisms.

Here, we have used 10 natural accessions of *Arabidopsis thaliana* that vary widely in their freezing tolerance and cold acclimation capacity, to characterize the natural variation in time-dependent responses of freezing tolerance, compatible solute content and expression of known cold regulated genes to deacclimation conditions. The data revealed that while solute and transcript levels declined sharply in all accessions after a shift of cold acclimated plants to ambient growth temperatures, freezing tolerance only declined in the more tolerant accessions, while more sensitive accessions were unaffected over the three day deacclimation period. In addition, correlations between freezing tolerance and some transcript and sugar levels that were highly significant in cold acclimated plants were lost during deacclimation, along with many correlations among transcript and solute levels.

## Results

### Deacclimation impacts freezing tolerance differently in natural accessions of *Arabidopsis thaliana*

**Figure 1:**
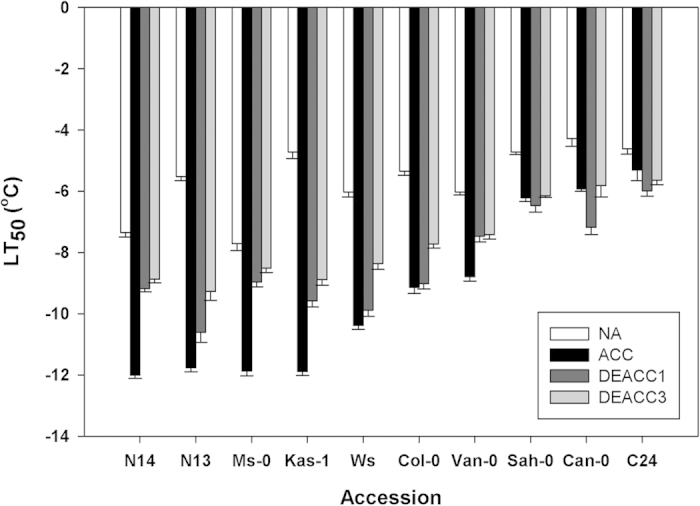
Freezing tolerance of leaves from 10 *Arabidopsis thaliana* accessions before (NA) and after (ACC) 14 d of cold acclimation at 4 °C and after 1 or 3 d of de-acclimation (DEACC1 and DEACC3) at 20 °C/18 °C day/night temperatures. Freezing tolerance is expressed as the LT_50_, the temperature that resulted in 50% ion leakage from the leaves. Bars represent the means ± SEM from five biological replicates, where each replicate comprised leaves from three plants. Accessions are ordered from the lowest LT_50_ after cold acclimation on the left to the highest on the right.

We determined the freezing tolerance of 10 natural accessions of Arabidopsis (Suppl. Table 1) before (non-acclimated; NA) and after 14 d of cold acclimation at 4 °C (acclimated; ACC), and after 1 d (DEACC1) and 3 d (DEACC3) of deacclimation at 20 °C/18 °C day/night temperature using an electrolyte leakage assay^24^. The resulting LT_50_ values are shown in Fig. 1. As reported previously^23^, all accessions showed increased freezing tolerance after cold acclimation, but acclimated freezing tolerance varied strongly among the accessions. Deacclimation resulted in reduced freezing tolerance in the majority of accessions, in particular during the first day. However, the three most sensitive accessions (Sah-0, Can-0 and C24) showed no reduction in their freezing tolerance over three days of deacclimation. In the more tolerant accessions, the strongest reduction of freezing tolerance was observed during the first day, while there was only a minor reduction in freezing tolerance between day 1 and day 3. However, this was not true for Ws and Col-0. In no case had the LT_50_ returned to the level of the non-acclimated plants after three days of deacclimation.

**Figure 2:**
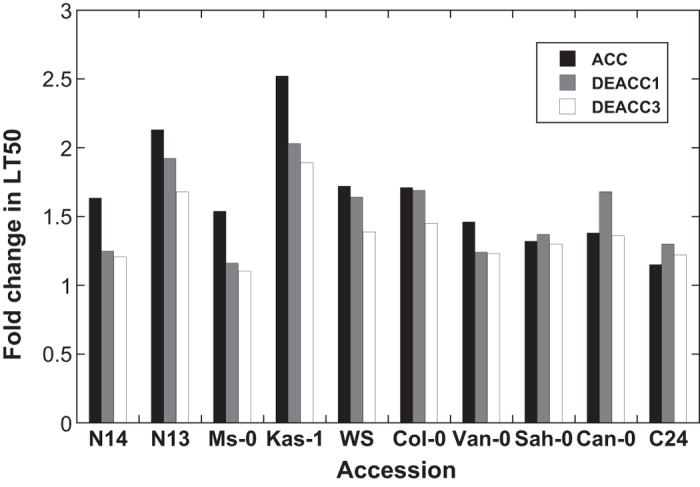
Effects of cold acclimation and subsequent deacclimation on relative freezing tolerance of 10 Arabidopsis accessions. Freezing tolerance after cold acclimation (ACC) and after deacclimation for 1 d (DEACC1) or 3 d (DEACC3) is expressed as the fold change in LT_50_ at these time points relative to the LT_50_ NA (compare Fig. 1).

The striking difference in the reaction of tolerant and sensitive accessions to deacclimation conditions is explored in more detail in Fig. 2. Here, we have calculated the fold change in LT_50_ values after cold acclimation and after deacclimation relative to the non-acclimated state to illustrate the decline in LT_50_ in a more comparable manner. This shows that while the tolerant accessions responded to warm temperature after cold acclimation strongly and quickly, the sensitive accessions showed almost no reaction over the investigated three day period, indicating natural genetic variability in the deacclimation response.

### Deacclimation is accompanied by a strong reduction in compatible solute content in all accessions

**Figure 3:**
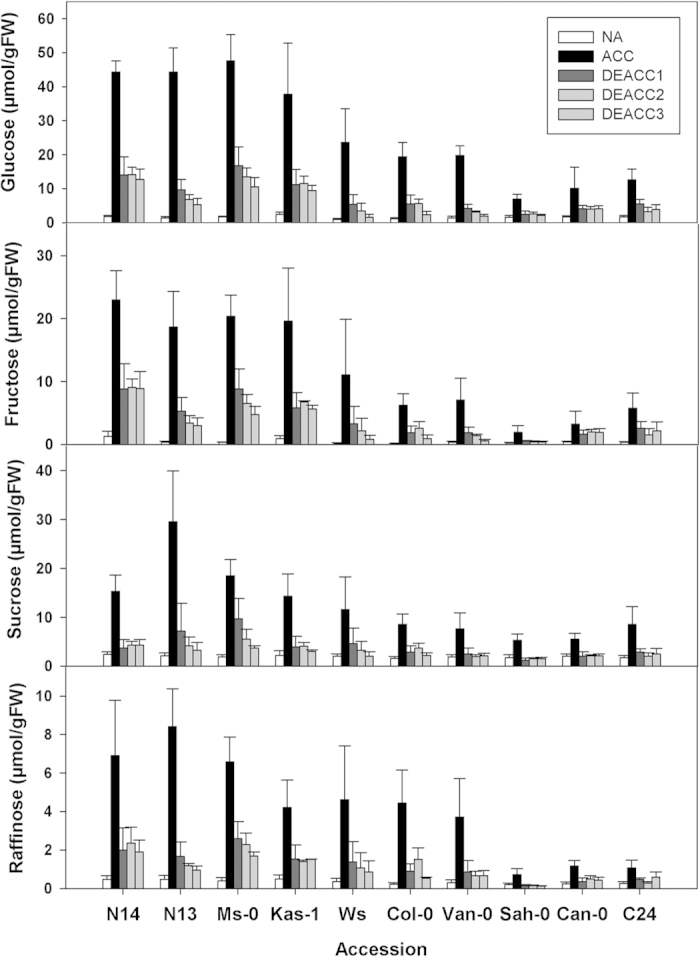
Content of soluble sugars in the rosettes of the 10 investigated *Arabidopsis* accessions. Plants were harvested before (NA) or after (ACC) 14 d of cold acclimation at 4 °C and after 1, 2 or 3 d of deacclimation (DEACC1, DEACC2 and DEACC3) at 20 °C/18 °C day/night temperatures. Accessions are ordered from the lowest LT_50_ after cold acclimation on the left to the highest on the right. Bars represent means ± SEM (n = 15).

**Figure 4:**
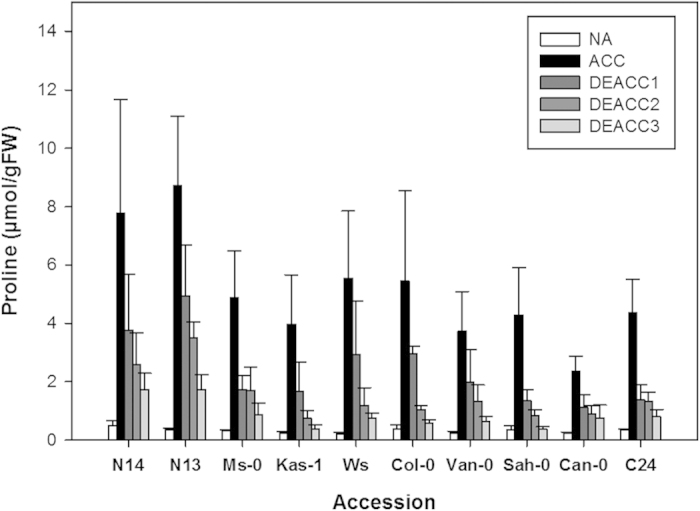
Proline content in the rosettes of the 10 investigated *Arabidopsis* accessions. Plants were harvested before (NA) or after (ACC) 14 d of cold acclimation and after 1, 2 or 3 d of deacclimation (DEACC1, DEACC2 and DEACC3) at 20 °C/18 °C day/night temperatures. Accessions are ordered from the lowest LT_50_ after cold acclimation on the left to the highest on the right. Bars represent means ± SEM (n = 15).

To further characterize the deacclimation response in the 10 accessions, we determined the content of Glc, Fru, Suc, Raf and Pro in non-acclimated plants (NA), after 14 d of cold acclimation (ACC) and after 1 d, 2 d and 3 d of deacclimation (DEACC1-3). In agreement with previous results^23^ all accessions showed increased sugar levels after cold acclimation (Fig. 3). The extent of sugar accumulation, however, was clearly higher in the tolerant than in the sensitive accessions. The levels of all four sugars declined dramatically during the first day of deacclimation and then either stayed constant or declined further over the next two days. A similar picture emerged for Pro content (Fig. 4). While sugar content declined back to the non-acclimated level in some cases, in particular for Suc, it remained generally elevated after 3 d of deacclimation for Pro. In contrast to what we observed for the LT_50_, the reduction in all five compatible solutes could be observed in both tolerant and sensitive accessions, indicating that compatible solute content and freezing tolerance became uncoupled during deacclimation.

### Cold regulated genes show complex expression patterns during deacclimation

**Figure 5:**
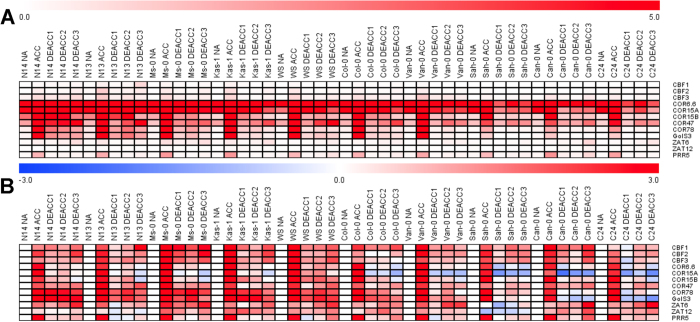
Expression of selected genes in 10 accessions before (NA) and after 14 d of cold acclimation at 4 °C (ACC) and after 1, 2 or 3 days of de acclimation (DEACC1, DEACC2 and DEACC3) at 20 °C/18 °C day/night temperatures. (**A**) Relative gene expression (2^−ΔCt^) as indicated by the different intensity of the red color and (**B**) log_2_ fold change in relative gene expression between the non-acclimated and cold acclimated or deacclimated plants on a scale from −3 (blue) to +3 (red). Accessions are ordered from the lowest LT_50_ after cold acclimation on the left to the highest on the right. The numerical values for 2^−ΔCt^ are given in Suppl. Table 1 and represent means from three replicate experiments.

To further characterize the reversibility of the cold response during deacclimation on the gene expression level, we determined the transcript abundance of 12 known cold induced genes^23^ before and after cold acclimation and after 1–3 d of deacclimation (Fig. 5, Suppl. Table 2). The highest transcript abundance under all conditions was found for *COR6.6* and *COR15A* and the lowest for the transcription factor genes *CBF1-3*, *ZAT6* and *ZAT12*. As expected for this selection of genes, they all showed strong cold induction in all accessions. During deacclimation, the transcript changes varied strongly among both the genes and the accessions. In general, the less freezing tolerant accessions showed stronger reductions in transcript abundance than the tolerant accessions (Fig. 5B), in clear contrast to the changes in LT_50_ (Fig. 2). In some cases, such as *COR15A, COR78* and *GolS3*, transcript levels in sensitive accessions were reduced below the levels under non-acclimated conditions, while *COR78* and *GolS3* transcript abundance remained at an elevated level even after 3 d of deacclimation in tolerant accessions (Fig. 5B). The expression of other genes, in particular the three *CBF* genes, showed a minimum after 1 d of deacclimation, but recovered at the later time points and remained higher after 3 d of deacclimation than under non-acclimating conditions.

### Correlation patterns among transcripts, compatible solutes and freezing tolerance change strongly during deacclimation

**Figure 6:**
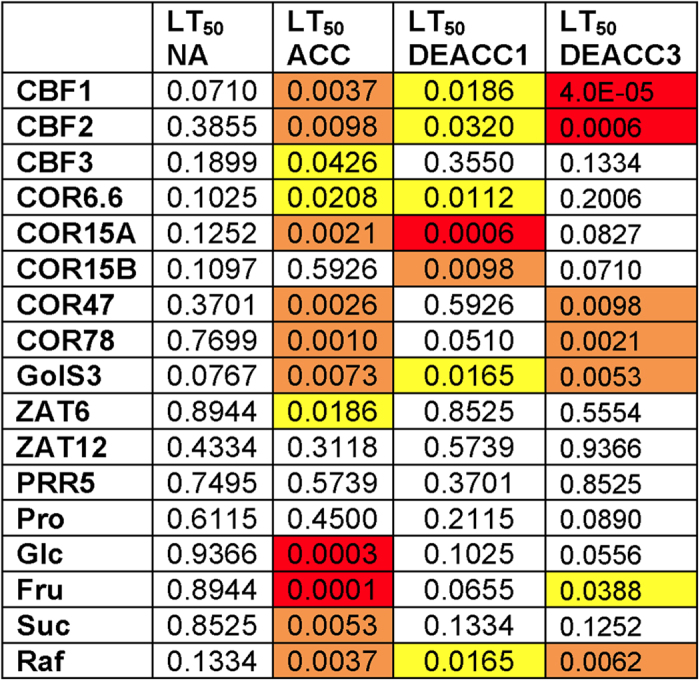
Correlation analysis between transcript abundance, sugar or proline content and freezing tolerance expressed as LT_50_. Plants were harvested before cold acclimation (non-acclimated, NA), after two weeks at 4 °C (cold acclimated, ACC), or after an additional one or three days of deacclimation (DEACC1 and DEACC3) at 20 °C/18 °C day/night temperature. The numbers indicate *P*-values from Spearman correlation analysis and significant correlations are color coded.

In agreement with a previous study on a larger set of accessions^23^, there were no significant (*P* < 0.05) correlations between LT_50_ NA and the content of the investigated solutes or transcripts for the 10 accessions investigated here (Fig. 6). On the other hand, the content of all four sugars and of several transcripts was significantly correlated with LT_50_ ACC. The number of correlations declined during deacclimation, but some correlations persisted, again indicating that the plants were not back to the non-acclimated state after 3 d of deacclimation. Quite strikingly, the expression of *CBF1* and *CBF2* remained correlated with LT_50_, while the expression of some CBF regulated genes such as *COR6.6* and *COR15A* was no longer correlated.

**Figure 7:**
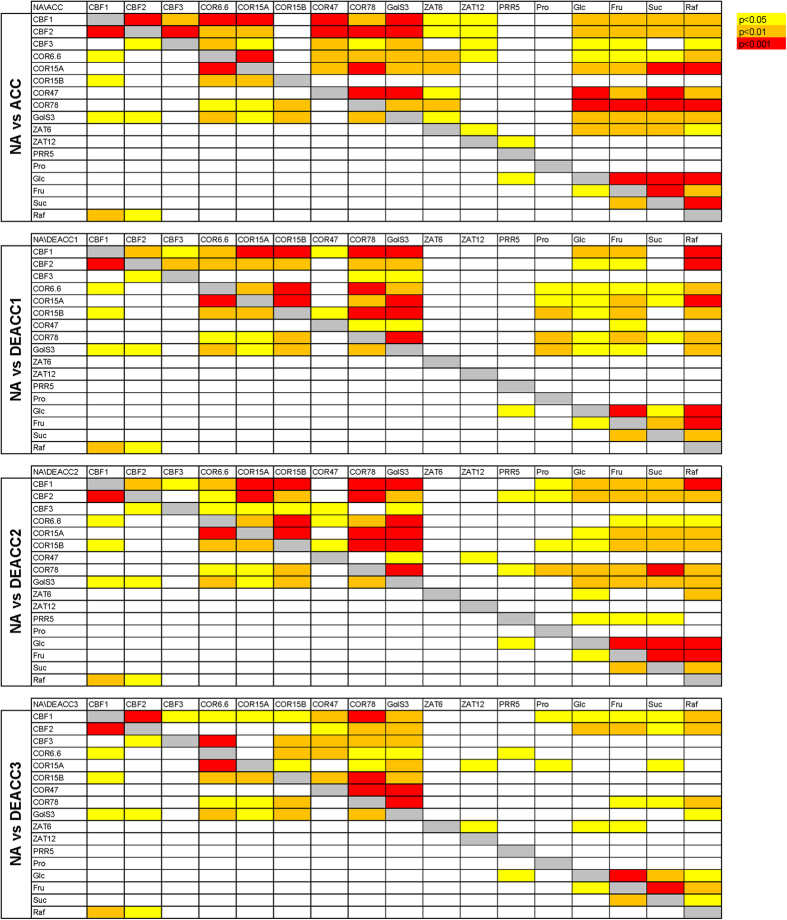
Correlation analysis between transcript abundance and sugar or proline content. Plants were harvested before cold acclimation (non-acclimated, NA), after two weeks at 4 °C (cold acclimated, ACC), or after an additional one, two or three days of deacclimation (DEACC1, DEACC2 and DEACC3) at 20 °C/18 °C day/night temperature. *P*-values from Spearman correlations were color coded as indicated. In each panel, the lower left half corresponds to the NA samples to allow direct comparison. The corresponding *P*- and r_s_-values can be found in Suppl. Tables 2 and 3, respectively.

A global correlation analysis among the content of the five solutes and the transcripts encoded by the 12 cold induced genes also indicated an incomplete reversal of cold acclimation after 3 d of deacclimation. For an easier overview, Fig. 7 only shows the color-coded *P*-value ranges of these correlations. The numerical *P*-values can be found in Suppl. Table 3 and the corresponding r_s_-values are given in Suppl. Table 4. While we detected mainly correlations among the *COR* and *GolS3* transcripts in the non-acclimated state, after cold acclimation these correlations were extended to more *COR* genes and also involved the *CBF* and *ZAT* genes. Further, the four sugars showed extensive correlations among each other and with the *CBF*, *GolS3* and almost all *COR* genes. The vast majority of these correlations were positive (Suppl. Table 4).

In particular, the correlations between sugar levels and the various transcript levels were strongly reduced during deacclimation, while the correlations between the transcript levels of the different *CBF* and *COR* genes were preserved to a much larger extent. The most notable exceptions were the many correlations of *ZAT6* and *ZAT12* transcripts with *CBF* and *COR* transcripts, which were already no longer significant after 1 d of deacclimation. On the other hand, several correlations, e.g. observed between the transcript levels of the *CBF1* and *CBF2* genes and expression of *GolS3* and Raf content remained significant throughout the 3 d deacclimation period. Taken together, these data suggest tightly controlled regulatory patterns that govern transcript and metabolite abundance during deacclimation.

## Discussion

While it is widely assumed that a shift of plants from cold to warm conditions results in a rapid loss of freezing tolerance^12^,^13^, our results reveal a surprisingly large natural variation in the deacclimation response of Arabidopsis. The tolerant accession Ms-0, for instance, had lost 81% of the additional freezing tolerance obtained during 14 d of cold acclimation after 3 d of deacclimation. On the other hand, Col-0 had lost only 37% of its cold-induced freezing tolerance and the sensitive accession Sah-0 none. A more rapid loss of freezing tolerance in more tolerant genotypes has been reported before for grape (*Vitis vinifera* L. and *V. labrusca* B.)^25^, *Hydrangea* spec.^26^,^27^, and white clover (*Trifolium repens* L.)^28^. However, for blueberry the opposite trend was found (*Vaccinium* spec.)^29^. Taken together, these data indicate that deacclimation is not just a passive reversal of cold acclimation, but rather a genetically determined process. In the future, this will allow the identification of genetic determinants of deacclimation through quantitative trait locus (QTL) mapping with recombinant inbred line (RIL) populations, derived from accessions differing in their acclimated freezing tolerance, that have already been successfully used to map freezing tolerance QTL^30^,^31^,^32^.

Metabolite profiling of Arabidopsis Col-0 revealed that the global composition of the metabolome after 24 h of deacclimation approached that of non-acclimated plants^33^. However, no further detailed analysis was provided. While positive correlations between the content of various compatible solutes and acclimated, but not non-acclimated, freezing tolerance have been found in many plant species (see^3^ for a review) and also for the four sugars investigated in the present study, these correlations were partially lost during deacclimation. The contents of the sugars and of proline were strongly reduced already after 1 d of deacclimation and unlike freezing tolerance, solute content decreased strongly also in sensitive accessions and reached non-acclimated levels in some accessions after 3 d of deacclimation. From the available data we can not exclude that sensitive accessions accumulate alternative compatible solutes during deacclimation. It has been shown previously that freezing tolerance is not dependent on the presence of a particular solute, such as Raf^34^. A general decrease in sugar content during deacclimation was also found in *Hydrangea*^26^,^27^, peach^35^, wheat and canola^36^, but no sufficient numbers of genotypes were investigated to allow a correlation analysis comparable to ours.

Changes in transcript levels of cold induced genes during deacclimation were more variable both among genes and among accessions. Most transcripts were more strongly reduced in abundance during deacclimation in sensitive than in tolerant accessions. A strong reduction in the abundance of transcripts of almost all cold induced genes during deacclimation has previously been shown for the accession Col-0^17^,^37^. Future proteomic studies will be needed to show, whether the observed reduction in transcript abundance leads to a corresponding reduction in the abundance of the encoded proteins, or whether the proteins are more stable. Since sugar content was strongly reduced in all accessions, the strong correlations between *COR* gene transcripts and sugar content were lost during deacclimation. Interestingly, most of the correlations between *CBF* and *COR* transcripts that were evident in the cold acclimated plants also persisted during deacclimation, indicating that the CBF regulon remained tightly coordinated during this process. On the metabolic level, this coordination extended from *CBF1* and *CBF2* through *GolS3* expression to the final product Raf. In addition, all steps of this pathway remained correlated with leaf freezing tolerance, indicating its central importance in the regulated decline in freezing tolerance during deacclimation. In striking contrast, transcript levels of *COR6.6* and *COR15A* were no longer correlated with LT_50_ after 3 d of deacclimation, while transcript levels of *COR47* and *COR78* remained correlated. A strongly reduced expression of the cold induced genes *COR15A*, *COR15B*, *COR47* and *GolS3* during deacclimation was also reported for Col-0 from microarray hybridization experiments^37^.

While gene expression data during deacclimation have rarely been reported, the content of cold induced dehydrin proteins during deacclimation has been reported in several studies. All three dehydrins investigated in blueberry^29^ and two investigated each in canola and wheat^36^ showed a strong reduction in their abundance during deacclimation. However, in two grass species only one of the two detected dehydrins showed this behavior, while the other was clearly induced during deacclimation^38^. In addition, the content of cold induced ice recrystallization inhibition proteins in the Antarctic hair grass (*Deschampsia antarctica*) decreased strongly during deacclimation, in parallel with the corresponding transcript levels^39^. These data indicate that deacclimation is accompanied by massive changes at all levels of plant metabolism, from gene expression to protein and metabolite content. The future challenge will be to elucidate how these changes are regulated and integrated to allow plants not only to reduce their freezing tolerance, but at the same time adapt to the increased temperature and resume growth and development.

In conclusion, the present study is the first to provide a systematic investigation into the natural variation of plant deacclimation on the phenotype, metabolite and transcript levels. It shows that deacclimation is a tightly regulated process with a strong genetic basis and thereby indicates the way for further genetic and molecular studies in this important and largely neglected area of plant-environment interactions.

## Methods

### Plant material

We used eight *Arabidopsis thaliana* accessions that were previously characterized for their acclimated and non-acclimated freezing tolerance as part of a larger population of 54 accessions^23^. In addition, we included the accessions Kashmir-1 (Kas-1) and Vancouver-0 (Van-0) in our experiments (see Suppl. Table 1 for details). Plants were grown in soil in a greenhouse at a temperature of 20 °C during the day, 18 °C during the night at 16 h day length at a minimum of 200 μE cm^−2^ s^−1^ until 42 days after sowing (non-acclimated plants). For cold acclimation, plants were transferred for an additional 14 days to a 4 °C growth cabinet at 16 h day length at 90 μE cm^−2^ s^−1^ (see^23^,^40^,^41^ for details). After 14 days of cold acclimation plants were transferred back to greenhouse conditions for one, two or three days of deacclimation. Three independent experiments were performed over a time span of several months, which lead to somewhat higher stadard errors in the metabolite measurements, due to differences in the light conditions in the greenhouse.

### Freezing experiments

Freezing damage was determined as electrolyte leakage after freezing of detached leaves to temperatures ranging from −1 °C to −22 °C at 4 °C/h and LT_50_ was calculated from the leakage values as described^24^.

### Sugar and proline analysis

Single rosettes were harvested from five different plants in each of three replicate experiments at mid-day (6 to 8 h after lights-on). Soluble sugars were extracted and quantified by high performance anion exchange chromatography as described previously^23^,^42^. Proline measurements were performed on the same leaf samples using a photometric assay^41^,^43^.

### qRT-PCR analysis of gene expression

Total RNA was extracted from rosette material pooled from five plants in each of three replicate experiments using either Trizol reagent (Invitrogen) or a lysis buffer containing 100 mM Tris-HCl (pH 8.5–9.0), 25 mM EDTA, 25 mM EGTA, 2% SDS, 100 mM ß-mercaptoethanol supplemented with phenol, chloroform and isoamylalcohol. RNA samples were DNase treated (Ambion, Austin, TX, USA, or ThermoFisher Scientific). RNA quality controls, first strand cDNA synthesis and cDNA quality controls were performed as described recently^23^. Quantitative PCR was performed with an ABI PRISM 7900 HT 384-well plate Sequence Detection System (Applied Biosystems, Darmstadt, Germany). Reactions containing 2.5 μl 2 × SYBR Green Master Mix (Fast Power SYBR Green; Applied Biosystems), 0.5 μl cDNA (diluted 5-fold) and 2 μl of 0.5 μM primers were pipetted using an Evolution P3 pipetting robot (PerkinElmer, Zaventem, Belgium). The sequences of all primers have been published^23^. Ct values for the genes of interest were normalized by subtracting the mean Ct of four reference genes^23^. All normalized expression values can be found in Suppl. Table 2.

### Computational methods

Spearman rank order correlation analysis was performed in R using the command *rcorr* from the package *Hmisc*.

## Additional Information

**How to cite this article**: Zuther, E. *et al.* Time-dependent deacclimation after cold acclimation in *Arabidopsis thaliana* accessions. *Sci. Rep.* **5**, 12199; doi: 10.1038/srep12199 (2015).

## Supplementary Material

Supplementary Information

Supplementary Table 2
