# Supplementary material for: Time-dependent deacclimation after cold acclimation in Arabidopsis thaliana accessions
Source: Sci Rep. 2015 Jul 15;5:12199. doi: 10.1038/srep12199 (PMC4648415; doi:10.1038/srep12199)
Supplement: Supplementary Information [file srep12199-s1.pdf]

Time-dependent deacclimation after cold acclimation in *Arabidopsis thaliana* accessions

Ellen Zuther[1], Ilona Juszczak[2,3], Yang Ping Lee[1,4], Margarete Baier[2] and Dirk K. Hincha[1]*

[1]Max-Planck-Institut für Molekulare Pflanzenphysiologie, Am Mühlenberg 1, D-14476 Potsdam, Germany

[2]FU Berlin, Institute of Biology, DCPS, Plant Physiology, Königin-Luise-Straße 12-16, 14195 Berlin, Germany

[3]present address: Universität Bonn, Institut für Molekulare Physiologie und Biotechnologie der Pflanzen, Kirschallee 1, D-53115 Bonn, Germany

[4]present address: FELDA Agricultural Services Sdn Bhd, Tingkat 7, Balai Felda, Jalan Gurney 1, 54000 Kuala Lumpur, Malaysia

*Author for correspondence:

Dirk K. Hincha, Max-Planck-Institut für Molekulare Pflanzenphysiologie, Am Mühlenberg 1, D-14476 Potsdam, Germany

Phone: +49 331 567 8253, Fax: +49 331 567 8250, email: hincha@mpimp-golm.mpg.de

Supplemental Material

Supplemental Table 1 (pdf). List of all accessions with information on their geographic origin and the INRA Versailles accession IDs.

Supplemental Table 2 (xls). Relative expression ($2^{-\Delta Ct}$) of all genes indicated in Fig. 5. Transcript abundance was determined by qRT-PCR and normalized to the transcript abundance of four reference genes. The data represent the means from three independent experiments.

Supplemental Table 3 (pdf). Spearman correlation matrix with the numerical values of the adjusted *P*-values of all pair-wise correlations for the expression of cold induced genes and the content of sugars and proline under non-acclimated conditions (lower left part of all panels) and after cold acclimation, and after subsequent deacclimation for 1, 2, or 3 days (upper right part of the respective panels). Significance levels of *P*-values are indicated by colors as in Fig. 7.

Supplemental Table 4 (pdf). Spearman correlation matrix with the numerical values of the correlation coefficients (r_S) of all pair-wise correlations for the expression of cold induced genes and the content of sugars and proline under non-acclimated conditions (lower left part of all panels) and after cold acclimation, and after subsequent deacclimation for 1, 2, or 3 days (upper right part of the respective panels). For all significant correlations (compare Fig. 7 and Suppl. Table 3), the direction (positive or negative) is indicated by the green or brown color.

Supplemental Table S1: List of all accessions with information on their geographic origin and the INRA Versailles accession IDs.

Abbreviated name	Name	Country	Latitude	Longitude	INRA accession ID
C24		POR	40.24	-8.42	183AV
Can-0	Canary Islands	ESP	28.00	-15.30	163AV
Col-0	Columbia	POL	52.73	15.15	186AV
Kas-1	Kashmir	IND	34.20	76.83	108AV
Ms-0	Moscow	RUS	55.75	37.35	93AV
N13	Konchezero	RUS	62.12	34.01	266AV
N14	Sampo Mountain	RUS	61.10	34.50	267AV
Sah-0	Sierra Alhambra	ESP	38.87	3.07	233AV
Van-0	Vancouver	CDN	49.27	-123.25	161AV
Ws	Wassilewskija	RUS	52.22	30.38	84AV

p<0.05
p<0.01
p<0.001

NA vs ACC

NA\ACC	CBF1	CBF2	CBF3	COR6.6	COR15A	COR15B	COR47	COR78	GolS3	ZAT6	ZAT12	PRR5	Pro	Glc	Fru	Suc	Raf
CBF1	NA	0.0002	0.0098	0.0000	0.0005	0.1334	0.0006	0.0010	0.0000	0.0128	0.0320	0.4500	0.5372	0.0037	0.0073	0.0026	0.0013
CBF2	0.0005	NA	0.0003	0.0017	0.0037	0.3118	0.0000	0.0008	0.0001	0.0208	0.0112	0.3259	0.5739	0.0017	0.0031	0.0031	0.0053
CBF3	0.0890	0.0353	NA	0.0085	0.0353	0.6500	0.0026	0.0260	0.0037	0.0655	0.0353	0.3701	0.4011	0.0165	0.0233	0.1025	0.0320
COR6.6	0.0112	0.0890	0.0956	NA	0.0008	0.1899	0.0085	0.0085	0.0017	0.0085	0.0388	0.3701	0.3403	0.0233	0.0353	0.0233	0.0098
COR15A	0.0890	0.2716	0.0604	0.0003	NA	0.1509	0.0013	0.0000	0.0062	0.0017	0.1334	0.7699	0.0827	0.0010	0.0026	0.0006	0.0008
COR15B	0.0467	0.1899	0.2229	0.0017	0.0031	NA	0.3550	0.1899	0.2345	0.8734	0.8944	0.0956	0.3403	0.4841	0.5554	0.1601	0.0956
COR47	0.0890	0.0710	0.6500	0.6500	0.7092	0.3550	NA	0.0000	0.0000	0.0233	0.0655	0.5372	0.3855	0.0002	0.0013	0.0008	0.0017
COR78	0.2345	0.2589	0.3550	0.0233	0.0388	0.0021	0.5739	NA	0.0010	0.0053	0.1601	0.7495	0.1334	0.0000	0.0002	0.0000	0.0001
GolS3	0.0186	0.0353	0.1509	0.0098	0.0289	0.0073	0.2229	0.0073	NA	0.0467	0.0655	0.4500	0.6115	0.0026	0.0073	0.0026	0.0013
ZAT6	0.2115	0.4669	0.4500	0.2115	0.4841	0.2847	0.3403	0.6696	0.7904	NA	0.0260	0.0710	0.2466	0.0017	0.0037	0.0085	0.0186
ZAT12	0.7904	0.6893	0.5192	0.4669	0.2345	0.8734	0.2716	0.7904	0.6115	0.1899	NA	0.0146	0.5739	0.0956	0.1420	0.1697	0.3259
PRR5	0.5015	0.5192	0.5372	0.7699	0.9155	0.8944	0.8944	0.2115	0.4334	0.7495	0.8110	NA	0.2229	0.3550	0.3701	0.6696	1.0000
Pro	0.6500	0.5926	0.5015	0.8317	0.2847	0.1899	1.0000	0.2115	0.6115	0.5739	0.9788	0.3550	NA	0.2981	0.3701	0.2466	0.0827
Glc	0.4171	0.9788	0.9788	0.7495	0.5372	0.5192	0.8734	0.8734	0.5926	0.2345	0.5015	0.0388	0.9788	NA	0.0000	0.0002	0.0003
Fru	0.4841	0.9788	0.6500	0.5739	0.6307	0.2716	0.5926	0.2847	0.9366	0.8317	0.7092	0.8944	0.2847	0.0388	NA	0.0006	0.0010
Suc	0.4171	0.7495	0.8525	0.7293	0.9577	0.2589	0.1173	0.1252	0.3550	0.6893	0.3118	0.3118	0.2229	0.4171	0.0045	NA	0.0002
Raf	0.0017	0.0353	0.3550	0.2981	0.4841	0.3259	0.2345	0.7904	0.2847	0.4841	0.9155	0.2589	0.6893	0.1025	0.1899	0.2345	NA

NA vs DEACC1

NA\DEACC1	CBF1	CBF2	CBF3	COR6.6	COR15A	COR15B	COR47	COR78	GolS3	ZAT6	ZAT12	PRR5	Pro	Glc	Fru	Suc	Raf
CBF1	NA	0.0010	0.0146	0.0021	0.0003	0.0001	0.0467	0.0000	0.0001	0.1334	0.6500	0.6500	0.0556	0.0062	0.0053	0.0710	0.0001
CBF2	0.0005	NA	0.0085	0.0085	0.0013	0.0098	0.4171	0.0037	0.0021	0.3855	0.5015	0.7092	0.0604	0.0128	0.0289	0.0655	0.0003
CBF3	0.0890	0.0353	NA	0.1797	0.0655	0.0510	0.1797	0.0233	0.0112	0.5554	0.1420	0.6893	0.0604	0.3550	0.3550	0.6307	0.0655
COR6.6	0.0112	0.0890	0.0956	NA	0.0053	0.0002	0.1601	0.0008	0.0013	0.3259	0.6115	0.8944	0.0233	0.0165	0.0260	0.0260	0.0037
COR15A	0.0890	0.2716	0.0604	0.0003	NA	0.0008	0.1797	0.0010	0.0005	0.7904	0.2345	0.7092	0.0320	0.0165	0.0073	0.0260	0.0002
COR15B	0.0467	0.1899	0.2229	0.0017	0.0031	NA	0.0388	0.0000	0.0000	0.3118	0.7293	0.5739	0.0053	0.0165	0.0098	0.0767	0.0021
COR47	0.0890	0.0710	0.6500	0.6500	0.7092	0.3550	NA	0.0208	0.0320	0.1252	0.6893	0.0956	0.1697	0.1420	0.0467	0.0827	0.0956
COR78	0.2345	0.2589	0.3550	0.0233	0.0388	0.0021	0.5739	NA	0.0000	0.2115	0.6307	0.3550	0.0031	0.0112	0.0098	0.0426	0.0013
GolS3	0.0186	0.0353	0.1509	0.0098	0.0289	0.0073	0.2229	0.0073	NA	0.3550	0.4669	0.4669	0.0062	0.0289	0.0208	0.0604	0.0021
ZAT6	0.2115	0.4669	0.4500	0.2115	0.4841	0.2847	0.3403	0.6696	0.7904	NA	0.1097	0.0890	0.8525	0.0556	0.1252	0.4669	0.1797
ZAT12	0.7904	0.6893	0.5192	0.4669	0.2345	0.8734	0.2716	0.7904	0.6115	0.1899	NA	0.5192	0.2716	0.8110	0.7699	0.8944	0.6500
PRR5	0.5015	0.5192	0.5372	0.7699	0.9155	0.8944	0.8944	0.2115	0.4334	0.7495	0.8110	NA	0.7092	0.8944	0.8944	0.8525	0.7495
Pro	0.6500	0.5926	0.5015	0.8317	0.2847	0.1899	1.0000	0.2115	0.6115	0.5739	0.9788	0.3550	NA	0.2115	0.1697	0.1899	0.1025
Glc	0.4171	0.9788	0.9788	0.7495	0.5372	0.5192	0.8734	0.8734	0.5926	0.2345	0.5015	0.0388	0.9788	NA	0.0000	0.0146	0.0001
Fru	0.4841	0.9788	0.6500	0.5739	0.6307	0.2716	0.5926	0.2847	0.9366	0.8317	0.7092	0.8944	0.2847	0.0388	NA	0.0045	0.0001
Suc	0.4171	0.7495	0.8525	0.7293	0.9577	0.2589	0.1173	0.1252	0.3550	0.6893	0.3118	0.3118	0.2229	0.4171	0.0045	NA	0.0085
Raf	0.0017	0.0353	0.3550	0.2981	0.4841	0.3259	0.2345	0.7904	0.2847	0.4841	0.9155	0.2589	0.6893	0.1025	0.1899	0.2345	NA

NA vs DEACC2

NA\DEACC2	CBF1	CBF2	CBF3	COR6.6	COR15A	COR15B	COR47	COR78	GolS3	ZAT6	ZAT12	PRR5	Pro	Glc	Fru	Suc	Raf
CBF1	NA	0.0010	0.0467	0.0045	0.0005	0.0002	0.2229	0.0002	0.0001	0.0510	0.8110	0.1509	0.0112	0.0031	0.0073	0.0085	0.0003
CBF2	0.0005	NA	0.0767	0.0128	0.0000	0.0045	0.2345	0.0000	0.0017	0.0556	0.5554	0.0112	0.0388	0.0037	0.0021	0.0053	0.0037
CBF3	0.0890	0.0353	NA	0.0233	0.0208	0.0233	0.0320	0.0710	0.0353	0.2006	0.7904	0.9366	0.2466	0.4669	0.5015	0.3855	0.1509
COR6.6	0.0112	0.0890	0.0956	NA	0.0045	0.0001	0.0467	0.0031	0.0002	0.3118	0.7495	0.3701	0.3118	0.0655	0.0320	0.0233	0.0146
COR15A	0.0890	0.2716	0.0604	0.0003	NA	0.0008	0.0767	0.0000	0.0001	0.0767	0.9577	0.0604	0.0655	0.0112	0.0098	0.0073	0.0037
COR15B	0.0467	0.1899	0.2229	0.0017	0.0031	NA	0.0208	0.0001	0.0000	0.1601	0.6500	0.1899	0.0467	0.0146	0.0053	0.0013	0.0026
COR47	0.0890	0.0710	0.6500	0.6500	0.7092	0.3550	NA	0.0604	0.0289	0.8110	0.0467	0.9366	0.4171	0.6307	0.2589	0.1697	0.2589
COR78	0.2345	0.2589	0.3550	0.0233	0.0388	0.0021	0.5739	NA	0.0000	0.1097	0.8734	0.0320	0.0098	0.0073	0.0010	0.0008	0.0021
GolS3	0.0186	0.0353	0.1509	0.0098	0.0289	0.0073	0.2229	0.0073	NA	0.1697	0.7293	0.1697	0.0556	0.0085	0.0037	0.0013	0.0017
ZAT6	0.2115	0.4669	0.4500	0.2115	0.4841	0.2847	0.3403	0.6696	0.7904	NA	0.7495	0.1509	0.2229	0.0186	0.0604	0.0827	0.0026
ZAT12	0.7904	0.6893	0.5192	0.4669	0.2345	0.8734	0.2716	0.7904	0.6115	0.1899	NA	0.6115	0.6115	0.7495	0.9788	0.6115	0.8944
PRR5	0.5015	0.5192	0.5372	0.7699	0.9155	0.8944	0.8944	0.2115	0.4334	0.7495	0.8110	NA	0.0827	0.0208	0.0165	0.0112	0.1252
Pro	0.6500	0.5926	0.5015	0.8317	0.2847	0.1899	1.0000	0.2115	0.6115	0.5739	0.9788	0.3550	NA	0.0956	0.0827	0.0655	0.0956
Glc	0.4171	0.9788	0.9788	0.7495	0.5372	0.5192	0.8734	0.8734	0.5926	0.2345	0.5015	0.0388	0.9788	NA	0.0000	0.0003	0.0002
Fru	0.4841	0.9788	0.6500	0.5739	0.6307	0.2716	0.5926	0.2847	0.9366	0.8317	0.7092	0.8944	0.2847	0.0388	NA	0.0000	0.0006
Suc	0.4171	0.7495	0.8525	0.7293	0.9577	0.2589	0.1173	0.1252	0.3550	0.6893	0.3118	0.3118	0.2229	0.4171	0.0045	NA	0.0026
Raf	0.0017	0.0353	0.3550	0.2981	0.4841	0.3259	0.2345	0.7904	0.2847	0.4841	0.9155	0.2589	0.6893	0.1025	0.1899	0.2345	NA

NA vs DEACC3

NA\DEACC3	CBF1	CBF2	CBF3	COR6.6	COR15A	COR15B	COR47	COR78	GolS3	ZAT6	ZAT12	PRR5	Pro	Glc	Fru	Suc	Raf
CBF1	NA	0.0000	0.0289	0.0426	0.0388	0.0165	0.0053	0.0001	0.0013	0.2589	1.0000	0.8734	0.0289	0.0146	0.0112	0.0426	0.0031
CBF2	0.0005	NA	0.1252	0.1420	0.0890	0.0556	0.0208	0.0017	0.0098	0.2115	0.6696	0.7092	0.1420	0.0098	0.0021	0.0165	0.0013
CBF3	0.0890	0.0353	NA	0.0005	0.2006	0.0013	0.0073	0.0045	0.0085	0.1420	0.9155	0.1252	0.1173	0.4011	0.2589	0.4669	0.0767
COR6.6	0.0112	0.0890	0.0956	NA	0.2006	0.0026	0.0062	0.0165	0.0208	0.5015	0.8944	0.0112	0.1797	0.6307	0.4841	0.6115	0.3701
COR15A	0.0890	0.2716	0.0604	0.0003	NA	0.0426	0.1173	0.0146	0.0098	0.6500	0.0146	0.9577	0.0165	0.2716	0.2847	0.0208	0.0767
COR15B	0.0467	0.1899	0.2229	0.0017	0.0031	NA	0.0021	0.0002	0.0031	0.5015	0.7495	0.1420	0.1252	0.2716	0.1420	0.1173	0.1025
COR47	0.0890	0.0710	0.6500	0.6500	0.7092	0.3550	NA	0.0003	0.0001	0.7092	0.9788	0.1509	0.4500	0.5372	0.1797	0.3259	0.0655
COR78	0.2345	0.2589	0.3550	0.0233	0.0388	0.0021	0.5739	NA	0.0001	0.3259	0.7699	0.5554	0.0767	0.0710	0.0208	0.0289	0.0045
GolS3	0.0186	0.0353	0.1509	0.0098	0.0289	0.0073	0.2229	0.0073	NA	0.7904	0.4171	0.4500	0.1173	0.3259	0.1334	0.1252	0.0146
ZAT6	0.2115	0.4669	0.4500	0.2115	0.4841	0.2847	0.3403	0.6696	0.7904	NA	0.0467	0.6307	0.6500	0.0388	0.0320	0.3259	0.0890
ZAT12	0.7904	0.6893	0.5192	0.4669	0.2345	0.8734	0.2716	0.7904	0.6115	0.1899	NA	0.8317	0.1601	0.6893	0.4841	0.5372	1.0000
PRR5	0.5015	0.5192	0.5372	0.7699	0.9155	0.8944	0.8944	0.2115	0.4334	0.7495	0.8110	NA	0.9788	0.2589	0.3118	0.2589	0.2589
Pro	0.6500	0.5926	0.5015	0.8317	0.2847	0.1899	1.0000	0.2115	0.6115	0.5739	0.9788	0.3550	NA	0.0604	0.3550	0.1797	0.1697
Glc	0.4171	0.9788	0.9788	0.7495	0.5372	0.5192	0.8734	0.8734	0.5926	0.2345	0.5015	0.0388	0.9788	NA	0.0002	0.0037	0.0233
Fru	0.4841	0.9788	0.6500	0.5739	0.6307	0.2716	0.5926	0.2847	0.9366	0.8317	0.7092	0.8944	0.2847	0.0388	NA	0.0008	0.0026
Suc	0.4171	0.7495	0.8525	0.7293	0.9577	0.2589	0.1173	0.1252	0.3550	0.6893	0.3118	0.3118	0.2229	0.4171	0.0045	NA	0.0146
Raf	0.0017	0.0353	0.3550	0.2981	0.4841	0.3259	0.2345	0.7904	0.2847	0.4841	0.9155	0.2589	0.6893	0.1025	0.1899	0.2345	NA

NA vs ACC

NA\ACC	CBF1	CBF2	CBF3	COR6.6	COR15A	COR15B	COR47	COR78	GolS3	ZAT6	ZAT12	PRR5	Pro	Glc	Fru	Suc	Raf
CBF1	NA	0.900	0.736	0.936	0.873	0.482	0.864	0.845	0.927	0.718	0.645	0.255	0.209	0.791	0.755	0.809	0.836
CBF2	0.873	NA	0.882	0.827	0.791	0.336	0.927	0.855	0.918	0.682	0.727	0.327	0.191	0.827	0.800	0.800	0.773
CBF3	0.536	0.636	NA	0.745	0.636	0.155	0.809	0.664	0.791	0.573	0.636	0.300	0.282	0.700	0.673	0.518	0.645
COR6.6	0.727	0.536	0.527	NA	0.855	0.427	0.745	0.745	0.827	0.745	0.627	0.300	0.318	0.673	0.636	0.673	0.736
COR15A	0.536	0.364	0.582	0.882	NA	0.464	0.836	0.936	0.764	0.827	0.482	0.100	0.545	0.845	0.809	0.864	0.855
COR15B	0.609	0.427	0.400	0.827	0.800	NA	0.309	0.427	0.391	0.055	0.045	-0.527	0.318	0.236	0.200	0.455	0.527
COR47	0.536	0.564	0.155	0.155	-0.127	0.309	NA	0.927	0.936	0.673	0.573	0.209	0.291	0.891	0.836	0.855	0.827
COR78	0.391	0.373	0.309	0.673	0.627	0.818	0.191	NA	0.845	0.773	0.455	0.109	0.482	0.936	0.900	0.964	0.918
GolS3	0.691	0.636	0.464	0.736	0.655	0.755	0.400	0.755	NA	0.609	0.573	0.255	0.173	0.809	0.755	0.809	0.836
ZAT6	0.409	0.245	-0.255	0.409	0.236	0.355	0.318	0.145	0.091	NA	0.664	0.564	0.382	0.827	0.791	0.745	0.691
ZAT12	0.091	0.136	0.218	0.245	0.391	-0.055	-0.364	-0.091	-0.173	0.427	NA	0.709	-0.191	0.527	0.473	0.445	0.327
PRR5	-0.227	-0.218	-0.209	0.100	0.036	0.045	-0.045	0.409	0.264	-0.109	-0.082	NA	-0.400	0.309	0.300	0.145	0.000
Pro	-0.155	-0.182	0.227	0.073	0.355	0.427	0.000	0.409	0.173	-0.191	-0.009	0.309	NA	0.345	0.300	0.382	0.545
Glc	0.273	0.009	-0.009	0.109	0.209	0.218	-0.055	-0.055	-0.182	0.391	0.227	-0.627	0.009	NA	0.982	0.900	0.882
Fru	0.236	0.009	0.155	0.191	0.164	0.364	0.182	0.355	0.027	0.073	-0.127	-0.045	0.355	0.627	NA	0.864	0.845
Suc	0.273	0.109	-0.064	0.118	0.018	0.373	0.500	0.491	0.309	0.136	-0.336	0.336	0.400	0.273	0.782	NA	0.900
Raf	0.827	0.636	0.309	0.345	0.236	0.327	0.391	0.091	0.355	0.236	-0.036	-0.373	-0.136	0.518	0.427	0.391	NA

positive correlation
negativ correlation

NA vs DEACC1

NA\DEACC1	CBF1	CBF2	CBF3	COR6.6	COR15A	COR15B	COR47	COR78	GolS3	ZAT6	ZAT12	PRR5	Pro	Glc	Fru	Suc	Raf
CBF1	NA	0.845	0.709	0.818	0.882	0.918	0.609	0.927	0.909	0.482	-0.155	0.155	0.591	0.764	0.773	0.564	0.918
CBF2	0.873	NA	0.745	0.745	0.836	0.736	0.273	0.791	0.818	0.291	-0.227	-0.127	0.582	0.718	0.655	0.573	0.882
CBF3	0.536	0.636	NA	0.436	0.573	0.600	0.436	0.673	0.727	0.200	-0.473	0.136	0.582	0.309	0.309	0.164	0.573
COR6.6	0.727	0.536	0.527	NA	0.773	0.900	0.455	0.855	0.836	0.327	0.173	0.045	0.673	0.700	0.664	0.664	0.791
COR15A	0.536	0.364	0.582	0.882	NA	0.855	0.436	0.845	0.873	0.091	-0.391	-0.127	0.645	0.700	0.755	0.664	0.891
COR15B	0.609	0.427	0.400	0.827	0.800	NA	0.627	0.955	0.945	0.336	-0.118	0.191	0.773	0.700	0.736	0.555	0.818
COR47	0.536	0.564	0.155	0.155	-0.127	0.309	NA	0.682	0.645	0.491	-0.136	0.527	0.445	0.473	0.609	0.545	0.527
COR78	0.391	0.373	0.309	0.673	0.627	0.818	0.191	NA	0.964	0.409	-0.164	0.309	0.800	0.727	0.736	0.618	0.836
GolS3	0.691	0.636	0.464	0.736	0.655	0.755	0.400	0.755	NA	0.309	-0.245	0.245	0.764	0.655	0.682	0.582	0.818
ZAT6	0.409	0.245	-0.255	0.409	0.236	0.355	0.318	0.145	0.091	NA	0.509	0.536	-0.064	0.591	0.491	0.245	0.436
ZAT12	0.091	0.136	0.218	0.245	0.391	-0.055	-0.364	-0.091	-0.173	0.427	NA	0.218	-0.364	0.082	-0.100	-0.045	-0.155
PRR5	-0.227	-0.218	-0.209	0.100	0.036	0.045	-0.045	0.409	0.264	-0.109	-0.082	NA	0.127	-0.045	-0.045	-0.064	-0.109
Pro	-0.155	-0.182	0.227	0.073	0.355	0.427	0.000	0.409	0.173	-0.191	-0.009	0.309	NA	0.409	0.445	0.427	0.518
Glc	0.273	0.009	-0.009	0.109	0.209	0.218	-0.055	-0.055	-0.182	0.391	0.227	-0.627	0.009	NA	0.945	0.709	0.909
Fru	0.236	0.009	0.155	0.191	0.164	0.364	0.182	0.355	0.027	0.073	-0.127	-0.045	0.355	0.627	NA	0.782	0.909
Suc	0.273	0.109	-0.064	0.118	0.018	0.373	0.500	0.491	0.309	0.136	-0.336	0.336	0.400	0.273	0.782	NA	0.745
Raf	0.827	0.636	0.309	0.345	0.236	0.327	0.391	0.091	0.355	0.236	-0.036	-0.373	-0.136	0.518	0.427	0.391	NA

NA vs DEACC2

NA\DEACC2	CBF1	CBF2	CBF3	COR6.6	COR15A	COR15B	COR47	COR78	GolS3	ZAT6	ZAT12	PRR5	Pro	Glc	Fru	Suc	Raf
CBF1	NA	0.845	0.609	0.782	0.873	0.891	0.400	0.900	0.918	0.600	0.082	-0.464	0.727	0.800	0.755	0.745	0.882
CBF2	0.873	NA	0.555	0.718	0.945	0.782	0.391	0.927	0.827	0.591	0.200	-0.727	0.627	0.791	0.818	0.773	0.791
CBF3	0.536	0.636	NA	0.673	0.682	0.673	0.645	0.564	0.636	0.418	-0.091	-0.027	0.382	0.245	0.227	0.291	0.464
COR6.6	0.727	0.536	0.527	NA	0.782	0.918	0.609	0.800	0.900	0.336	0.109	-0.300	0.336	0.573	0.645	0.673	0.709
COR15A	0.536	0.364	0.582	0.882	NA	0.855	0.555	0.927	0.909	0.555	0.018	-0.582	0.573	0.727	0.736	0.755	0.791
COR15B	0.609	0.427	0.400	0.827	0.800	NA	0.682	0.918	0.982	0.455	-0.155	-0.427	0.609	0.709	0.773	0.836	0.809
COR47	0.536	0.564	0.155	0.155	-0.127	0.309	NA	0.582	0.655	0.082	-0.609	0.027	0.273	0.164	0.373	0.445	0.373
COR78	0.391	0.373	0.309	0.673	0.627	0.818	0.191	NA	0.936	0.509	-0.055	-0.645	0.736	0.755	0.845	0.855	0.818
GolS3	0.691	0.636	0.464	0.736	0.655	0.755	0.400	0.755	NA	0.445	-0.118	-0.445	0.591	0.745	0.791	0.836	0.827
ZAT6	0.409	0.245	-0.255	0.409	0.236	0.355	0.318	0.145	0.091	NA	0.109	-0.464	0.400	0.691	0.582	0.545	0.809
ZAT12	0.091	0.136	0.218	0.245	0.391	-0.055	-0.364	-0.091	-0.173	0.427	NA	-0.173	-0.173	0.109	-0.009	-0.173	0.045
PRR5	-0.227	-0.218	-0.209	0.100	0.036	0.045	-0.045	0.409	0.264	-0.109	-0.082	NA	-0.545	-0.682	-0.700	-0.727	-0.491
Pro	-0.155	-0.182	0.227	0.073	0.355	0.427	0.000	0.409	0.173	-0.191	-0.009	0.309	NA	0.527	0.545	0.573	0.527
Glc	0.273	0.009	-0.009	0.109	0.209	0.218	-0.055	-0.055	-0.182	0.391	0.227	-0.627	0.009	NA	0.927	0.882	0.891
Fru	0.236	0.009	0.155	0.191	0.164	0.364	0.182	0.355	0.027	0.073	-0.127	-0.045	0.355	0.627	NA	0.945	0.864
Suc	0.273	0.109	-0.064	0.118	0.018	0.373	0.500	0.491	0.309	0.136	-0.336	0.336	0.400	0.273	0.782	NA	0.809
Raf	0.827	0.636	0.309	0.345	0.236	0.327	0.391	0.091	0.355	0.236	-0.036	-0.373	-0.136	0.518	0.427	0.391	NA

NA vs DEACC3

NA\DEACC3	CBF1	CBF2	CBF3	COR6.6	COR15A	COR15B	COR47	COR78	GolS3	ZAT6	ZAT12	PRR5	Pro	Glc	Fru	Suc	Raf
CBF1	NA	0.936	0.655	0.618	0.627	0.700	0.773	0.909	0.836	0.373	0.000	0.055	0.655	0.709	0.727	0.618	0.800
CBF2	0.873	NA	0.491	0.473	0.536	0.591	0.682	0.827	0.736	0.409	0.145	-0.127	0.473	0.736	0.818	0.700	0.836
CBF3	0.536	0.636	NA	0.873	0.418	0.836	0.755	0.782	0.745	0.473	0.036	0.491	0.500	0.282	0.373	0.245	0.555
COR6.6	0.727	0.536	0.527	NA	0.418	0.809	0.764	0.700	0.682	0.227	0.045	0.727	0.436	0.164	0.236	0.173	0.300
COR15A	0.536	0.364	0.582	0.882	NA	0.618	0.500	0.709	0.736	-0.155	-0.709	-0.018	0.700	0.364	0.355	0.682	0.555
COR15B	0.609	0.427	0.400	0.827	0.800	NA	0.818	0.900	0.800	0.227	-0.109	0.473	0.491	0.364	0.473	0.500	0.518
COR47	0.536	0.564	0.155	0.155	-0.127	0.309	NA	0.882	0.918	0.127	0.009	0.464	0.255	0.209	0.436	0.327	0.573
COR78	0.391	0.373	0.309	0.673	0.627	0.818	0.191	NA	0.918	0.327	-0.100	0.200	0.555	0.564	0.682	0.655	0.782
GolS3	0.691	0.636	0.464	0.736	0.655	0.755	0.400	0.755	NA	0.091	-0.273	0.255	0.500	0.327	0.482	0.491	0.709
ZAT6	0.409	0.245	-0.255	0.409	0.236	0.355	0.318	0.145	0.091	NA	0.609	-0.164	0.155	0.627	0.645	0.327	0.536
ZAT12	0.091	0.136	0.218	0.245	0.391	-0.055	-0.364	-0.091	-0.173	0.427	NA	0.073	-0.455	0.136	0.236	-0.209	0.000
PRR5	-0.227	-0.218	-0.209	0.100	0.036	0.045	-0.045	0.409	0.264	-0.109	-0.082	NA	0.009	-0.373	-0.336	-0.373	-0.373
Pro	-0.155	-0.182	0.227	0.073	0.355	0.427	0.000	0.409	0.173	-0.191	-0.009	0.309	NA	0.582	0.309	0.436	0.445
Glc	0.273	0.009	-0.009	0.109	0.209	0.218	-0.055	-0.055	-0.182	0.391	0.227	-0.627	0.009	NA	0.891	0.791	0.673
Fru	0.236	0.009	0.155	0.191	0.164	0.364	0.182	0.355	0.027	0.073	-0.127	-0.045	0.355	0.627	NA	0.855	0.809
Suc	0.273	0.109	-0.064	0.118	0.018	0.373	0.500	0.491	0.309	0.136	-0.336	0.336	0.400	0.273	0.782	NA	0.709
Raf	0.827	0.636	0.309	0.345	0.236	0.327	0.391	0.091	0.355	0.236	-0.036	-0.373	-0.136	0.518	0.427	0.391	NA
